# A Bibliometric Analysis of Music's Role in Promoting Well-Being in Health Science Research

**DOI:** 10.12688/f1000research.168772.1

**Published:** 2025-09-04

**Authors:** Kanjanee Phanphairoj, Sutthisan Chumwichan, Ratsiri Thato, Dneya Udtaisuk, Debby Syahru Romadlon, Faizul Hasan

**Affiliations:** 1 Faculty of Nursing, Chulalongkorn University, Bangkok, Bangkok, Thailand; 2 Multidisciplinary approaches to improve holistic well-being for all ages: Harmonizing nursing science and music Research Group, Chulalongkorn University, Bangkok, Bangkok, Thailand; 3 Invited Researcher, Faculty of Education, Chulalongkorn University, Bangkok, Bangkok, Thailand; 4 Faculty of Education, Chulalongkorn University, Bangkok, Bangkok, Thailand

**Keywords:** music therapy, mental health promotion, neuroscience of music, healthy aging rehabilitation, and auditory health prevention.

## Abstract

**Background:**

The relationship between music and well-being has gained significant scholarly interest due to its implications for mental health, rehabilitation, and quality of life. Despite growing research, a large-scale bibliometric analysis capturing global trends, key contributors, and emerging themes in this interdisciplinary field remains unexplored. This study addresses this gap by systematically mapping the scientific landscape of music and well-being research over time, identifying dominant themes, and highlighting areas requiring further investigation.

**Methods:**

We conducted a bibliometric review of 16,585 peer-reviewed articles indexed in Scopus, spanning multiple disciplines, including psychology, medicine, and neuroscience. Using advanced computational methods—co-occurrence network analysis and Louvain clustering—we identified major research clusters and analyzed publication trends, geographic contributions, and evolving topics.

**Results:**

Five key thematic clusters emerged: (1) Well-being and Spiritual Growth, (2) Music Therapy for Anxiety and Pain, (3) Emotion and Cognition, (4) Rehabilitation in Older Adults, and (5) Hearing Health in Youth. Publication output increased sharply after 2018, with Music Therapy for Anxiety and Pain (Cluster 2) representing the largest share of research. The United States and European countries were the most prolific contributors, whereas regions with rich musical traditions, such as Africa and South Asia, were underrepresented. Emerging trends included neuroscientific explorations of music’s effects and digital therapeutic innovations (e.g., AI-driven music interventions). However, challenges in clinical implementation persist, including limited healthcare integration and insufficient training for practitioners, particularly nurses, who play a vital role in therapy delivery.

**Conclusions:**

This study offers a comprehensive foundation for advancing research on music and well-being, emphasizing the need for cross-cultural studies, deeper mechanistic insights, and ethical frameworks for digital applications. Future work should prioritize translating research into practice, ensuring equitable global representation, and addressing implementation barriers in healthcare settings.

## 1. Introduction

Music is a universal human experience that engages fundamental emotional and cognitive processes. ^1^ Historically recognized as both an art form and therapeutic tool, music exerts profound psychological and physiological effects, ^2, 3^ The music-wellbeing relationship has attracted growing interdisciplinary research attention across psychology, neuroscience, public health, and music therapy, ^4, 5^ Robust evidence demonstrates music’s capacity to enhance mood, reduce stress, improve cognition, strengthen social bonds, and complement mental and physical health interventions, ^6, 7^

Research on music and well-being has grown increasingly complex and multidisciplinary as scientific interest expands. ^8^ Studies of music therapy now encompass diverse settings including hospital wards, rehabilitation centers, classrooms, and community spaces ^8, 9^ Contemporary evidence positions music not as a passive pastime, but as an active modulator of health and emotional states. ^10^ Despite extensive research, the field lacks an integrated framework to elucidate its intellectual progression, key contributors, and thematic evolution.

Bibliometric analysis offers a robust quantitative framework for evaluating complex research landscapes. ^11^ By examining citation networks, publication patterns, and keyword co-occurrence, this method maps knowledge evolution while identifying pivotal studies and emerging frontiers. ^12^ Unlike traditional reviews, bibliometric synthesis creates conceptual integration that reveals an academic discipline’s structural development. ^13^

This study conducted a comprehensive bibliometric analysis of global research exploring the music-wellbeing relationship. Our analysis maps scientific developments while identifying: (1) key contributors and institutions, (2) dominant thematic clusters, and (3) emerging research trajectories in this interdisciplinary field.

## 2. Methodology

### 2.1 Data collection

**Figure 1.**
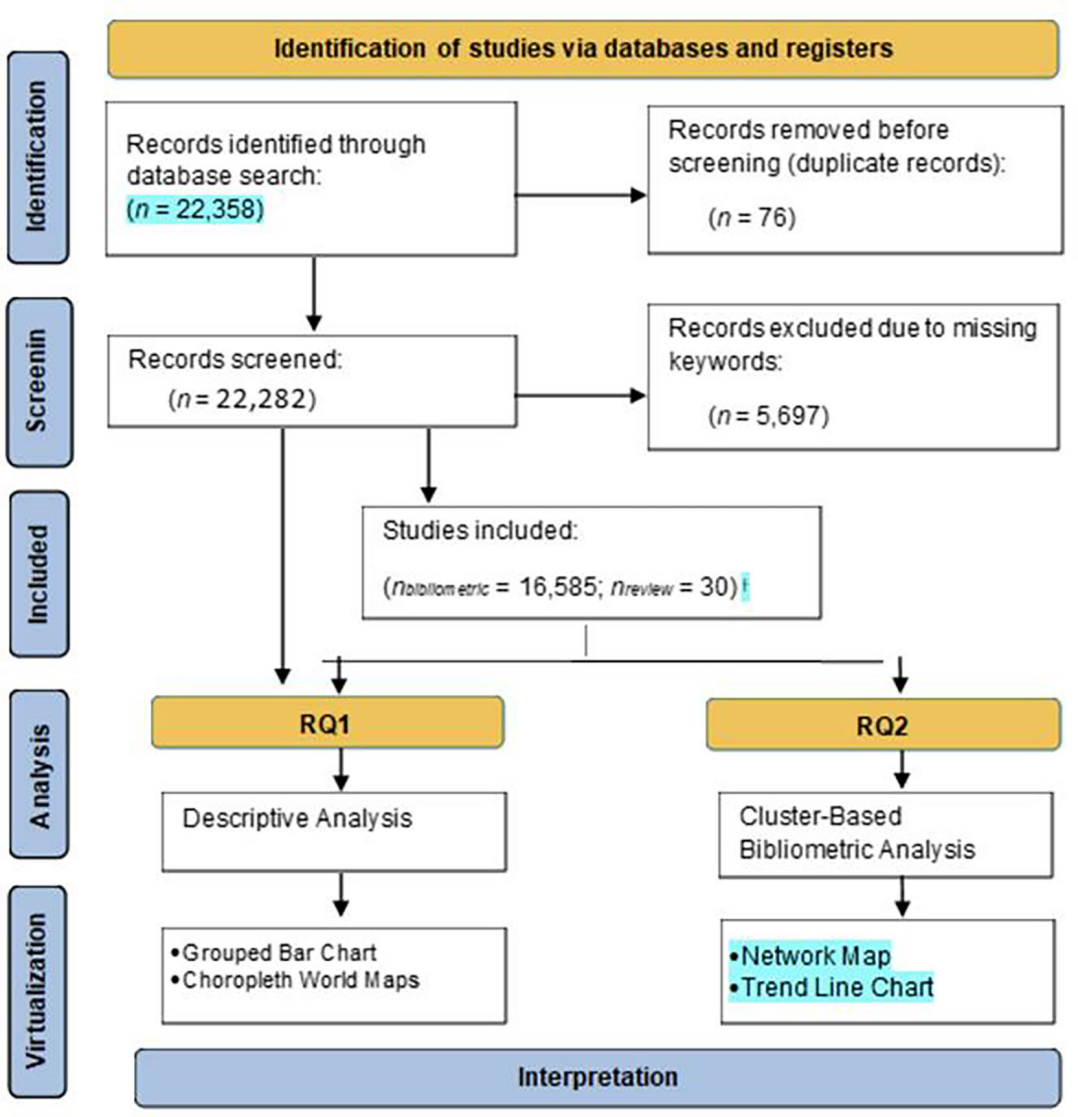
PRISMA flow diagram and methods (Adapted from Dzhunushalieva & Teuber, 2024 and Page et al., 2021). ^†^ Number of literature reviewed ( *n*_review_) was determined based on the balance of cosine similarities, piecewise regression by era, citation metrics, and thematic alignment of keywords.

This study followed the PRISMA 2020 framework ^14^ to ensure transparent data collection and screening processes ( Figure 1). We retrieved bibliographic records from Scopus, selected for its robust coverage of both music-related research and health sciences literature. On 21 February 2025, we developed a comprehensive search strategy targeting titles, abstracts, and keywords related to various well-being constructs. Our search methodology employed an iterative approach: first identifying root terms (e.g., “music,” “wellbeing”) and their frequently co-occurring keywords through preliminary analysis, then manually screening these terms to establish our final keyword set. The search was limited to English-language journal articles with no publication date restrictions and was last updated on 22 May 2025 (Appendix A). From an initial pool of 22,358 records, we included 16,585 publications for bibliometric analysis and selected 30 key articles for systematic review.

### 2.2 Data cleaning

We implemented a structured data cleaning pipeline using Python 3.13.3 to standardize terminology and improve keyword consistency. Our processing workflow incorporated pandas ^15^ for data manipulation and regular expressions for text normalization, supplemented by NLTK ^16^ for lemmatization. To address terminological variations, we developed a custom synonym dictionary that consolidated equivalent terms (e.g., mapping “covid” and “coronavirus” to “covid-19”). The research team manually reviewed and corrected lemmatization artifacts (e.g., converting “social medium” to “social media”). Final keyword selection prioritized the most frequently occurring representative term from each conceptual group.

### 2.3 Analysis and virtualization

We conducted descriptive analyses using R version 4.4.3 with the bibliometrix package. ^17^ The dataset was transformed into a bibliographic dataframe to examine key metrics including publication volume, document age, citation rates, and author productivity across tourism, marketing, and business management domains. Temporal publication trends were analyzed using Piecewise Linear Regression to identify inflection points in research output. For comparative visualization, we employed Python to generate figures illustrating both national-level productivity and international collaboration networks, enabling cross-field comparisons.

The clustering analysis employed a two-phase methodology. First, we constructed a keyword co-occurrence network using VOSviewer 1.6.20, ^18^ with nodes representing keywords and edges reflecting co-occurrence strength. We implemented Louvain clustering ^19– 21^ rather than the default method to better identify modular communities. The algorithm was executed 100 times with varying random seeds (1-100), with manual selection of the optimal solution based on alignment with expected thematic structures. In the second phase, we mapped articles to clusters using cosine similarity between article keyword vectors and cluster profiles. This approach, which considers both keyword composition and directional relationships, ^11, 22^ provided more accurate thematic alignment than frequency-based methods by capturing deeper semantic connections while avoiding overrepresentation of generic terms.

The cluster interpretation process combined quantitative bibliometric mapping with qualitative analysis through a structured three-phase approach. Following the scanning, sensing, and substantiating framework, ^23^ we first identified preliminary themes by examining high-frequency keywords and article titles. Next, we analyzed representative abstracts and author keywords to discern conceptual relationships. Finally, we triangulated these findings with citation metrics, temporal trends, and disciplinary discourse to validate themes. Cluster labels emerged inductively through synthesis of dominant keywords, metadata patterns, and content analysis of representative publications.

To enhance rigor, we incorporated an iterative reflection process using ChatGPT-4, which improved thematic clarity and visual coherence while maintaining human oversight. This approach aligns with: (1) contemporary bibliometric practices emphasizing quantitative-qualitative integration, ^22, 24^; (2) emerging LLM-assisted methodologies for complex data analysis ^19^; and (3) transdisciplinary bibliometric principles that facilitate cross-domain interpretation. ^25^

## 3. Results

### 3.1 Descriptive

Our temporal analysis using Piecewise Linear Regression identified four distinct research eras demarcated by inflection points at 1982.7, 2002.5, and 2017.5. The earliest period (Era1: 1898–1982) showed gradual growth with 0.31 publications/year, followed by accelerated output during Era2 (1983–2002; 8.03 publications/year). The field entered rapid expansion in Era3 (2003–2017; 50.46 publications/year), culminating in exponential growth in Era4 (2018–2024; 174.86 publications/year), as evidenced by the model’s exceptional fit (R ^2^ = .9962). Publication volume surged from 361 documents in Era1 to 11,681 in Era4 ( Table 1, Figure 3), paralleled by expansions in journal diversity, authorship networks, and keyword variety. While citation impact peaked in Era2 (37.93 citations/doc) and Era3 (37.18), Era4 showed reduced but still active engagement (9.92 citations/doc; 1.911 citations/year/doc). The field demonstrated progressive globalization through rising co-authorship (mean authors/doc: 1.2→4.6) and international collaboration (12%→43% of publications), reflecting its maturation into an interdisciplinary research domain.

**Table 1.** Bibliometric summary by era.

Timespan	Era1	Era2	Era3	Era4
Sources (Journals)	220	965	3096	3817
Documents	361	1,902	8,317	11,681
Annual Growth Rate %	4.26	11.20	10.19	2.20 ^†^
Document Average Age	56.10	29.70	13.30	3.27 ^†^
Average citations per doc	15.90	37.93	37.18	9.919 ^†^
Average citations per year per doc	0.28	1.33	2.63	1.911 ^†^
References	2,181	38,826	295,236	520,434
*DOCUMENT TYPES*				
Article	360	1746	7008	10174
Review	1	156	1309	1507
*DOCUMENT CONTENTS*				
Keywords Plus (ID)	1,110	4,770	16,428	21,198
Author’s Keywords (DE)	219	1,420	13,486	22,210
*AUTHORS*				
Authors	543	3,464	20,804	35,561
Author Appearances	589	3,928	26,796	49,308
Authors of single-authored docs	198	848	1,954	1,695
*AUTHORS COLLABORATION*				
Single-authored docs	211	931	2,199	1,926
Documents per Author	.67	.55	.40	.328
Co-Authors per Doc	1.63	2.07	3.22	4.22
International co-authorships %	1.39	5.99	17.82	23.62

Note. **Era1** = 1898–1982; **Era2** = 1983–2002; **Era3** = 2003–2017; **Era4** = 2018–2024.

^†^ As of 2024: Annual Growth Rate = 12.24%, Document Average Age = 3.55, Average Citations per Document = 10.72, Average Citations per Year per Document = 2.051.

**Figure 2.**
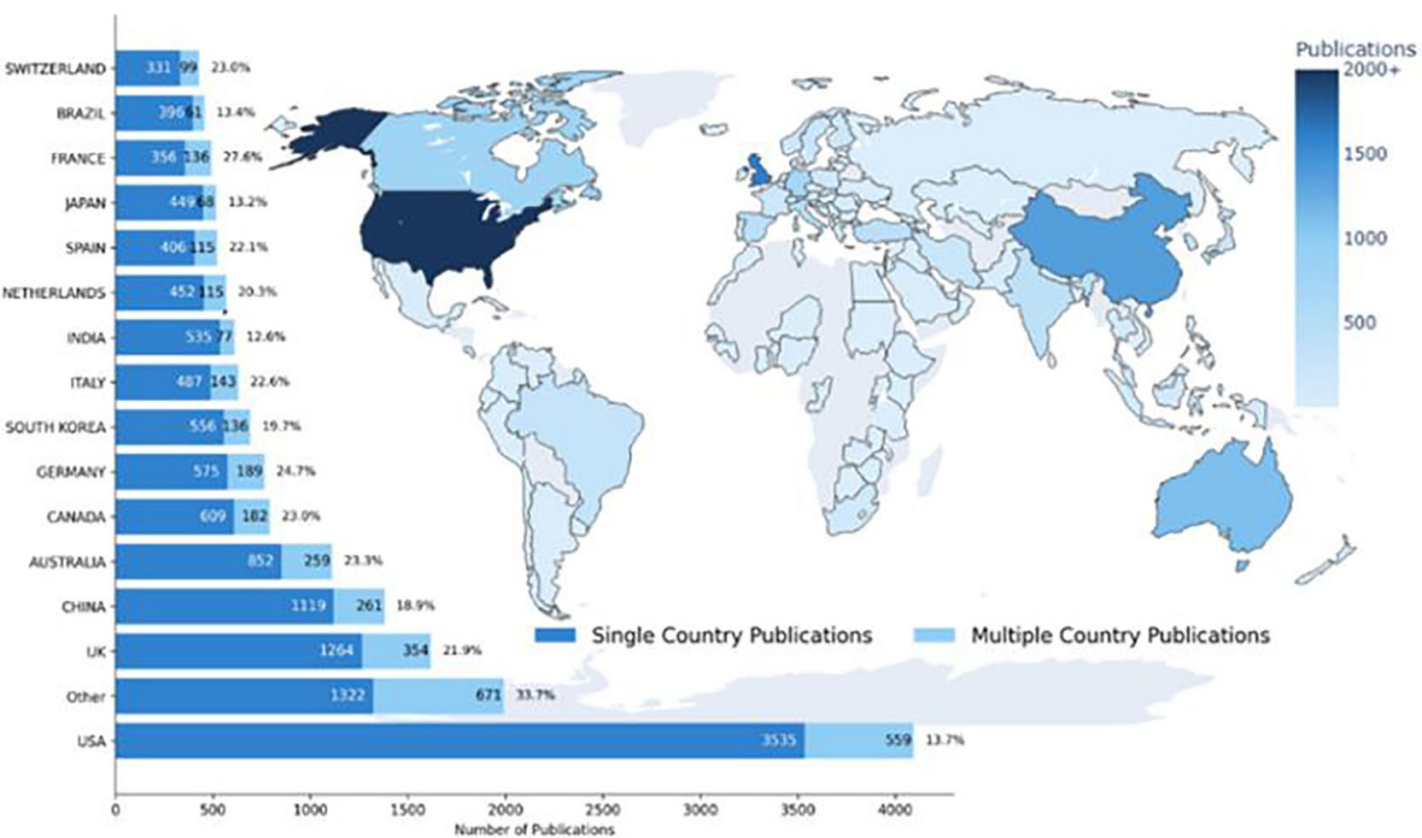
Global distribution of publications by corresponding author and collaboration type. Note. Bar chart and world map show the number of publications per country based on corresponding author counts.

**Figure 3.**
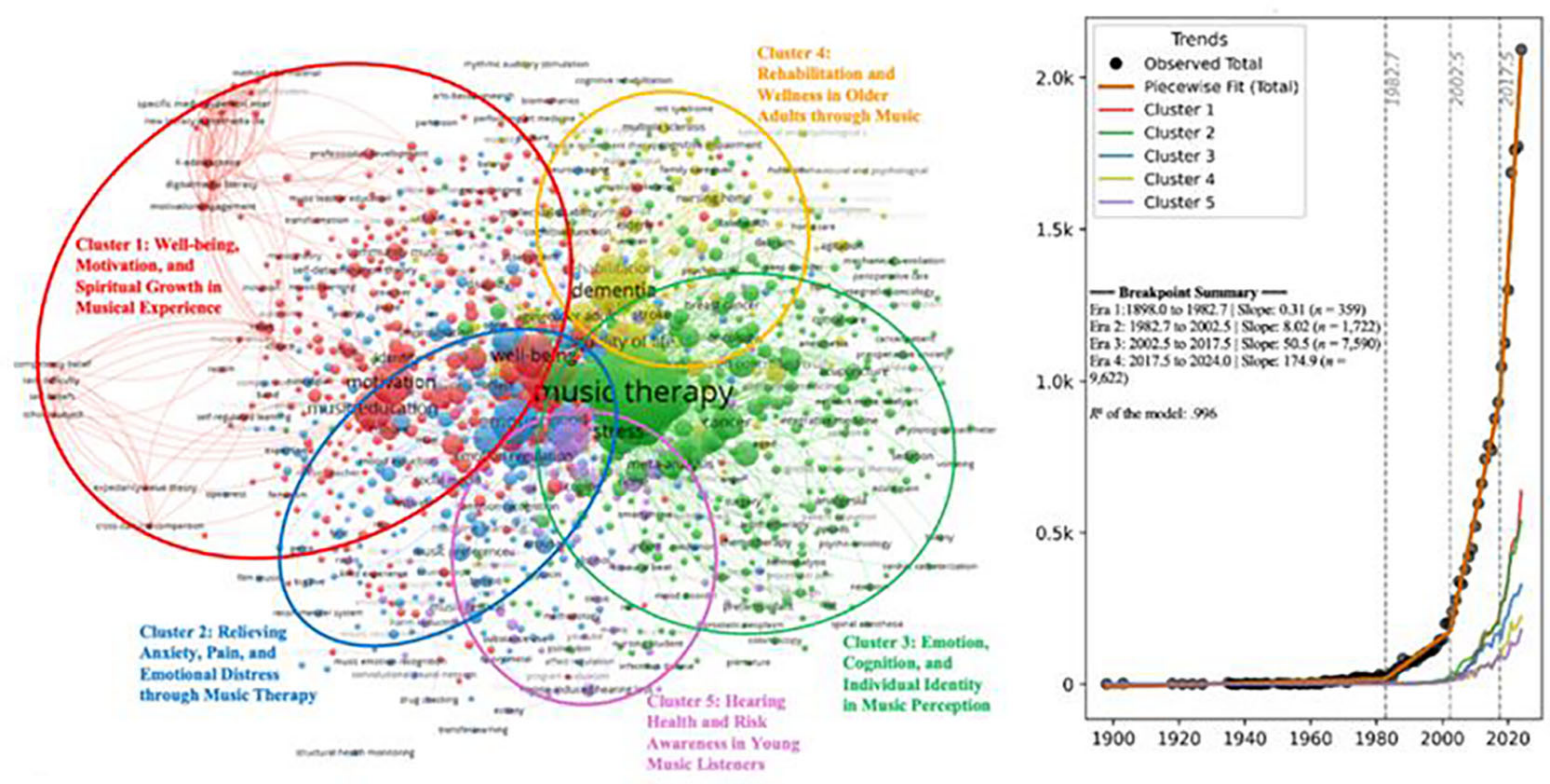
Music and well-being research clusters (left) and annual trend analysis with piecewise regression (right).

Beyond overall publication growth, our country-level analysis reveals significant variations in research output and collaborative patterns. The United States dominates with 4,094 publications, substantially outpacing the United Kingdom (1,618) and China (1,380). Other key contributors include Australia (1,111), Canada (791), and Germany (701). Notably, while the United States and India maintain high publication volumes, they demonstrate relatively limited international collaboration (13.6% and 12.1% respectively). This contrasts sharply with nations like South Korea (29.8% internationally co-authored publications), Italy (28.0%), and the Netherlands (25.7%), which show stronger global research integration. These disparities highlight fundamental differences in both productivity and scholarly connectivity among leading nations, as visually summarized in Figure 2. The findings suggest that research volume alone does not necessarily correlate with international engagement, revealing distinct national approaches to music and wellbeing research.

### 3.2 Cluster-based bibliometric analysis

Our Louvain clustering analysis of author keyword co-occurrence networks identified five distinct thematic research clusters ( Figure 3, left). Using cosine similarity, we mapped these cluster-level keyword profiles to individual articles, enabling longitudinal tracking of thematic evolution. Two clusters showed particularly rapid growth during 2010–2024: Cluster 1 (Well-being, Motivation, and Spiritual Growth in Musical Experience) and Cluster 2 (Music Therapy for Anxiety, Pain, and Emotional Distress). Cluster 3 (Music Perception: Emotion, Cognition, and Identity) demonstrated steady expansion, while Clusters 4 (Music-based Rehabilitation and Wellness in Older Adults) and 5 (Hearing Health and Risk Awareness in Youth) maintained consistent annual growth. These trends collectively highlight the field’s growing emphasis on mental health applications, aging populations, and auditory well-being ( Figure 3, right), reflecting broader societal health priorities in music research.

**Cluster 1: Well-being, Motivation, and Spiritual Growth in Musical Experience**

This cluster demonstrates music’s capacity to facilitate personal transformation through emotional enrichment, existential exploration, and identity development in both individual and communal contexts. The research reveals two key dimensions of musical experience: (1) an innate psychobiological foundation, evidenced by early work showing humans’ natural ability to synchronize emotional states with musical expressions, ^26^ and (2) culturally-mediated transformative effects. Neurochemical studies elucidate music’s ability to stimulate motivational and social bonding systems, enhancing psychological resilience. ^27^ In collective settings like raves, music facilitates symbolic healing through altered states of consciousness and emotional catharsis. ^28^ Active musical engagement—including singing, rapping, and movement—promotes self-actualization by fostering creativity, self-esteem, and identity consolidation. ^6^ Awe-inducing musical experiences further contribute to spiritual growth and meaning-making, ^29^ while intercultural musical practices strengthen community belonging and cross-cultural understanding. ^30^ Despite its transformative potential, this research stream remains under-cited relative to clinical applications, suggesting opportunities for greater scholarly recognition.

**Cluster 2: Relieving Anxiety, Pain, and Emotional Distress through Music Therapy**

This cluster examines the evidence-based application of music therapy for alleviating psychological and physical distress in clinical environments. While music’s calming effects have been historically acknowledged, structured clinical research only emerged in the 2000s through systematic reviews and controlled trials. A seminal review established that audio-delivered music interventions in hospital settings consistently reduce anxiety and improve mood, though physiological outcomes show more variability. ^31^ The evidence base has since expanded significantly, with clinical guidelines now incorporating music therapy for breast cancer care—both for general stress reduction ^32^ and specifically during chemotherapy to enhance emotional well-being and treatment tolerance. ^33^ Comparable therapeutic benefits have been documented in perinatal care, where music interventions decrease maternal anxiety and labor pain while improving birth experiences. ^34^ Comprehensive meta-analyses confirm these effects, demonstrating music therapy’s robust capacity to mitigate stress, regulate physiological arousal, and promote emotional balance across diverse clinical populations, ^35^ with additional benefits for fatigue management and sleep quality. ^36^ This progression from anecdotal use to evidence-based practice reflects the field’s maturation into a standardized therapeutic modality.

**Cluster 3: Emotion, Cognition, and Individual Identity in Music Perception**

This cluster elucidates the complex interplay between music perception, emotional processing, and cognitive functioning. Foundational research established that listeners demonstrate remarkable consistency in interpreting musical meaning through structured descriptors, indicating that musical expressiveness derives from shared symbolic and rhythmic conventions rather than subjective interpretation alone. ^37^ Neuroscientific evidence reveals music’s capacity to activate primal emotional networks, triggering memory recall, mood modulation, and physiological responses (e.g., chills)—findings that support evolutionary theories of music as a communication and social bonding mechanism. ^38^ Theoretical models have since delineated six distinct pathways for musical emotion induction (brainstem reflexes, conditioning, emotional contagion, visual imagery, episodic memory, and cognitive expectancy), emphasizing the need for mechanism-specific investigations. ^39^ Neurochemical research has further demonstrated dopamine-mediated effects on musical pleasure and motivation, providing direct evidence of music’s reward system engagement. ^40^ Contemporary work bifurcates into two productive directions: (1) applied research on music-based emotional regulation through personalized interventions, ^41^ and (2) fundamental studies of music’s cognitive effects, including neural plasticity and therapeutic applications across brain functions. ^42^ Together, these advances underscore music’s dual role as both an emotional modulator and cognitive stimulant.

**Cluster 4: Rehabilitation and Wellness in Older Adults through Music**

This cluster examines the evidence-based application of music therapy for geriatric rehabilitation, with particular focus on Parkinson’s disease and dementia populations. While initial research recognized music’s general calming effects, systematic therapeutic applications only gained empirical support in the 2000s. For Parkinson’s patients, active music therapy combining singing and rhythmic movement demonstrates significant improvements in motor symptoms (e.g., bradykinesia), emotional well-being, and functional capacity. ^43^ Complementary approaches using dance and rhythmic auditory stimulation enhance gait rehabilitation while simultaneously addressing cognitive-motor integration. ^44^ Emerging digital modalities show promise, as evidenced by a pilot study where virtual group music therapy reduced apathy and depression with strong participant adherence. ^45^ In dementia care, meta-analyses confirm music therapy’s efficacy in reducing behavioral symptoms (agitation, anxiety) and improving cognitive metrics and quality of life, ^46, 47^ often through multimodal interventions incorporating movement. Recent technological advances have expanded therapeutic access through ICT-based programs that manage neuropsychiatric symptoms via interactive digital platforms. ^48^ These developments reflect music therapy’s evolution from adjunctive comfort measure to standardized rehabilitation component in geriatric care.

**Cluster 5: Hearing Health and Risk Awareness in Young Music Listeners**

This cluster investigates hearing health risks associated with recreational music exposure among youth populations. Pioneering experimental studies first established the physiological vulnerability to high-decibel stimuli and fundamental mechanisms of auditory conditioning. ^49^ Clinical research subsequently identified subclinical auditory pathologies—including hyperacusis and tinnitus—in young music listeners without measurable hearing loss. ^50^ Public health studies have since shifted focus to behavioral prevention, with systematic reviews identifying personal audio devices and live music venues as primary risk factors for noise-induced hearing loss (NIHL) and advocating for targeted education initiatives, ^51, 52^ Emerging evidence reveals early-stage auditory damage in young musicians through advanced diagnostics, suggesting conventional audiometry may underestimate music-related hearing impairment. ^53^ While current rehabilitation options remain limited, novel interventions including gene therapy and precision medicine show promise for addressing irreversible auditory damage, ^54^ highlighting the need for both preventive strategies and advanced treatment development.

Following the scanning, sensing, and substantiating framework, ^23^ Table 2 presents seminal articles representative of each thematic cluster, identified through cosine similarity-based keyword alignment. The selected publications are organized chronologically by research era, showcasing influential works that exemplify each cluster’s conceptual focus. Citation metrics—including total citations (TC) and citations per year (CPY)—provide quantitative indicators of their sustained scholarly impact and temporal influence within the field.

**Table 2.** Representative articles by cluster and era with citation metrics.

Era	Year	Article Title	CPY	TC
**Cluster 1: Well-being, Motivation, and Spiritual Growth in Musical Experience**				
1	1983	The relationship between ratings of the emotional characters of musics and the **emotions aroused** by them	0.07	3
2	2000	The rave: **Spiritual healing** in modern western subcultures	3.69	96
3	2013	The **neurochemistry** of music	48.38	629
4	2021	How Do Music Activities Affect Health and **Well**- **Being**? A Scoping Review of Studies Examining Psychosocial Mechanisms	21.60	108
4	2023	Awe as a Pathway to **Mental and Physical Health**	21.33	64
4	2024	Music, **social cohesion**, and **intercultural understanding**: A conceptual framework for intercultural music engagement	5.00	10
**Cluster 2: Relieving Anxiety, Pain, and Emotional Distress through Music Therapy**				
2	2002	The effectiveness of music as an intervention for **hospital patients**: A systematic review	10.29	247
3	2017	Clinical practice guidelines on the evidence-based use of integrative therapies during and after **breast cancer treatment**	63.67	573
4	2020	Virtual reality and music therapy as distraction interventions to **alleviate anxiety and improve mood** states in **breast cancer** patients during chemotherapy	28.33	170
4	2020	Effects of music interventions on **stress-related outcomes**: a systematic review and two meta-analyses	42.33	254
4	2022	Music therapy for **stress reduction**: a systematic review and meta-analysis	46.00	184
4	2024	The role and outcomes of music therapy during **pregnancy**: a systematic review of randomized controlled trials	6.50	13
**Cluster 3: Emotion, Cognition, and Individual Identity in Music Perception**				
1	1935	**Expression in music**: a discussion of experimental studies and theories	1.87	170
2	2002	**Emotional sounds and the brain**: The neuro-affective foundations of musical appreciation	13.83	332
3	2008	**Emotional responses to music**: The need to consider underlying mechanisms	68.17	1,227
4	2019	**Dopamine modulates** the reward experiences elicited by music	31.71	222
4	2023	An **emotion-based personalized** music recommendation framework for emotion improvement	14.67	44
4	2024	The transformative power of music: Insights into **neuroplasticity**, health, and disease	7.50	15
**Cluster 4: Rehabilitation and Wellness in Older Adults through Music**				
2	2000	Active music therapy in **Parkinson’s** disease: An integrative method for motor and emotional rehabilitation	12.62	328
3	2017	Systematic review of systematic reviews of non-pharmacological interventions to treat behavioural disturbances in **older patients** with **dementia**. the SENATOR-OnTop series	39.33	354
4	2019	Music therapy and dance as gait rehabilitation in patients with **Parkinson** disease: A Review of Evidence	11.86	83
4	2020	Music therapy in the treatment of **dementia**: A systematic review and meta-analysis	23.00	138
4	2023	The effectiveness of non-pharmacological interventions using information and communication technologies for behavioral and psychological symptoms of **dementia**: A systematic review and meta-analysis	9.00	27
4	2024	Virtual group music therapy for apathy in **Parkinson’s** disease: A pilot study	5.50	11
**Cluster 5: Hearing Health and Risk Awareness in Young Music Listeners**				
1	1977	Galvanic skin response-orienting reflex and semantic conditioning and generalization with different unconditioned stimuli	0.29	14
2	1999	**Hypersensitivity** to sound. Questionnaire data, audiometry and classification	7.89	213
3	2007	**Noise** and hearing loss: A review	15.32	291
4	2020	**Inner ear gene therapies** take off: Current promises and future challenges	15.50	93
4	2021	Loud music and leisure noise is a common cause of chronic **hearing loss**, tinnitus and hyperacusis	9.80	49
4	2024	A longitudinal study investigating the effects of **noise exposure** on behavioural, electrophysiological and self-report measures of hearing in musicians with normal audiometric thresholds	2.00	4

**Note.** *CPY* = Citations Per Year, *TC* = Total Citations.

## Discussion

This bibliometric analysis reveals a rapidly expanding field of music and well-being research, characterized by distinct thematic clusters and evolving temporal trends. The dramatic surge in publications (2018-2024) reflects growing interdisciplinary recognition of music’s role in health and emotional wellness. The dominance of clinical-therapeutic applications (Cluster 2) aligns with global healthcare shifts toward non-pharmacological interventions, particularly for anxiety, pain management, and mood regulation, ^32, 35^ While declining citation rates in recent years may reflect either delayed impact assessment or thematic saturation, they underscore the need for innovative methodologies and deeper mechanistic investigations. Notably, the geographic distribution of research output shows disproportionate representation from Western nations (U.S. and Europe) compared to musically rich but academically underrepresented regions (Africa, South America), suggesting potential cultural biases that warrant systematic examination. ^55^ These findings collectively highlight both the field’s maturation and persistent gaps requiring cross-cultural, translational, and methodological advancements.

The prominence of Cluster 1 (Well-being and Spiritual Growth) and Cluster 3 (Emotion and Cognition) underscores music’s dual capacity as both a transformative cultural practice and a neurobiological modulator. Cluster 1’s focus on music’s ability to induce awe, foster meaning-making, and shape identity, ^29, 30^ resonates with contemporary positive psychology frameworks emphasizing transcendent experiences. Concurrently, Cluster 3’s neuroscientific advances have delineated music’s dopaminergic reward mechanisms ^40^ and emotion regulatory functions, ^41^ bridging aesthetic appreciation with empirical validation. The relatively lower citation impact of Cluster 1 may reflect an academic bias favoring quantitative clinical studies over qualitative or cross-cultural investigations of musical experience, suggesting the need for more pluralistic evaluation frameworks. While emerging digital therapeutics—including AI-driven music therapy and personalized recommendation systems ^6^—offer promising scalability, they raise unresolved ethical questions regarding equitable access and the preservation of musical-cultural authenticity that demand urgent scholarly attention.

Cluster 4 (Rehabilitation in Older Adults) and Cluster 5 (Hearing Health) address critical public health priorities across the lifespan. Cluster 4 demonstrates compelling evidence for music therapy’s efficacy in managing neurodegenerative conditions, ^46, 47^ yet its clinical integration remains inconsistent due to variability in therapeutic protocols and healthcare policy implementation. Conversely, Cluster 5 reveals a persistent prevention gap in youth hearing conservation, where established behavioral interventions like volume regulation and auditory education remain underutilized despite documented risks, ^52, 53^ While emerging biotechnological approaches (e.g., gene therapy for hearing restoration ^54^) offer promising avenues, their clinical translation requires rigorous evaluation to assess practical applicability and long-term outcomes. Together, these clusters underscore the need for both improved implementation of existing evidence-based music interventions and advancement of novel therapeutic solutions.

Nurses serve as pivotal implementers and advocates for music therapy integration in clinical care, leveraging their frontline position to address patients’ psychosocial needs through music-based interventions, ^36, 56, 57^ As primary caregivers, nurses are uniquely positioned to administer and evaluate music therapy applications for stress alleviation, pain reduction, and emotional support. Empirical studies document significant improvements in patient outcomes when nurses incorporate music therapy modalities—including structured listening sessions, live musical interactions, and personalized playlists—with demonstrated benefits for anxiety reduction, sleep improvement, and overall care satisfaction. ^58^ Particularly in palliative, dementia, and postoperative care settings, nurse-led music interventions have proven effective in enhancing communication, reducing agitation, and promoting relaxation when pharmacological options are limited. ^59^ However, persistent barriers including time limitations, inadequate training, and restrictive institutional policies continue to hinder broader implementation. To optimize patient outcomes, healthcare systems should: (1) incorporate music therapy competencies into nursing education curricula, (2) foster structured collaboration between nurses and certified music therapists, and (3) integrate evidence-based music interventions into standardized care protocols across clinical specialties.

While this study provides a comprehensive mapping of the music and well-being research landscape, several limitations warrant consideration. First, our Scopus-derived dataset, though extensive, may underrepresent non-English publications and gray literature, potentially overlooking culturally-specific music therapy applications. Second, while bibliometric analysis effectively reveals quantitative patterns, it cannot capture nuanced qualitative insights—a gap best addressed through complementary systematic reviews. These limitations suggest three critical directions for future research: (1) mechanistic studies examining longitudinal intervention effects and individual response variability, (2) ethical analyses of digital music therapy implementation, and (3) cross-cultural collaborations to diversify the evidence base by incorporating global musical traditions. Addressing these priorities will advance the field toward more inclusive, empirically-grounded applications of music for holistic health promotion and human flourishing.

## Conclusion

This bibliometric analysis emphasizes the swift expansion and interdisciplinary character of research concerning music and well-being, identifying five principal thematic clusters that highlight music’s therapeutic, cognitive, and emotional advantages. Although therapeutic applications predominate in the literature, burgeoning fields like as neuroscientific mechanisms, digital remedies, and cross-cultural studies offer exciting prospects for future investigation. Despite the field’s growth, differences in worldwide contributions and implementation gaps in healthcare settings—especially in nursing practice—necessitate enhanced inclusivity, standardized protocols, and translational research. Confronting these obstacles is essential for optimizing music’s capacity to improve well-being among varied populations and promoting evidence-based, scalable therapies.

## Data Availability

No data are associated with this article. Supplementary materials are available in the supplementary data. Zenodo: A Bibliometric Analysis of Music’s Role in Promoting Well-Being in Health Science Research, https://doi.org/10.5281/zenodo.16892715. ^60^ This project contains the following extended data: Appendix A Bibliometric Search Strategy.docx Data are available under the terms of the Creative Commons Attribution 4.0 International license (CC-BY 4.0).
