# Supplementary material for: A Bibliometric Analysis of Music's Role in Promoting Well-Being in Health Science Research
Source: F1000Res. 2026 May 26;14:873. Originally published 2025 Sep 4. [Version 3] doi: 10.12688/f1000research.168772.3 (PMC13309773; doi:10.12688/f1000research.168772.3)
Supplement: Peer review history

# Reviewer report: Reviewer response for version 3

**DOI:** 10.5256/f1000research.201300.r494057

**Published:** 2026-07-14

**Authors:** Diah Ratnawati

**Affiliations:** 1 Departmentof Community Health Nursing, Universitas Pembangunan Nasional Veteran Jakarta, South Jakarta, Jakarta, Indonesia

This work offers a pertinent and significant bibliometric analysis of the swiftly expanding interdisciplinary domain of music and well-being research. The study's methodological approach is a notable strength, utilizing a big dataset of over 16,000 articles and employing advanced techniques such as Louvain clustering, cosine similarity mapping, and piecewise linear regression. The finding of five thematic clusters offers a coherent and valuable intellectual framework for the discipline, and the discussion effectively links these findings to essential domains such as nursing practice and healthcare policy. This topic holds significant interest and relevance for the audience of F1000Research.

Is the work clearly and accurately presented and does it cite the current literature?

Yes

If applicable, is the statistical analysis and its interpretation appropriate?

Yes

Are all the source data underlying the results available to ensure full reproducibility?

Yes

Is the study design appropriate and is the work technically sound?

Yes

Are the conclusions drawn adequately supported by the results?

Yes

Are sufficient details of methods and analysis provided to allow replication by others?

Yes

Reviewer Expertise:

nursing, community nursing, gerontology, Cross-cultural community nursing and implementation research, Family, school, and community-based health promotion, Gerontic and family health.

I confirm that I have read this submission and believe that I have an appropriate level of expertise to confirm that it is of an acceptable scientific standard.

# Reviewer report: Reviewer response for version 3

**DOI:** 10.5256/f1000research.201300.r494054

**Published:** 2026-06-27

**Authors:** Vivi Leona Amelia

**Affiliations:** 1 Department of Nursing, Faculty of Health Sciences, Universitas Muhammadiyah Purwokerto, Purwokerto, Central Java, Indonesia

Detailed Responses to the Review Questions

 1. Is the work clearly and accurately presented and does it cite the current literature? — **Partly**

 The literature is current, relevant, and appropriately covers both methodological references (bibliometric guidelines, PRISMA, the analysis tools) and substantive references within each thematic cluster. The thematic presentation is coherent and accessible.

 However, accuracy is compromised in two specific places that **must be addressed**:

- **Copy-paste error in Section 2.3.** The Methods state that the dataset was examined for productivity "across tourism, marketing, and business management domains." This is unrelated to music and well-being and appears carried over from the authors' own prior bibliometric study (reference 61, Chumwichan & Meesing, 2025, on tourism/marketing/business management). This sentence must be rewritten to describe the actual domains of this study (e.g., psychology, medicine, neuroscience, music therapy). Its presence raises concern about how carefully the methods text was adapted, so the authors should also re-check the surrounding text for further carry-over.
- **Inconsistent framing of the 30-article component.** The abstract calls this a "targeted review of 30 key articles," while the Methods and Results refer to a "systematic review." These are not equivalent, and the inconsistency should be resolved (see Question 2).

To address this question, the authors should correct the domain error, harmonize the terminology describing the 30 articles across abstract, methods, and results, and verify that no other text has been inadvertently imported from prior work.

 2. Is the study design appropriate and is the work technically sound? — **Partly**

 The bibliometric design is appropriate, and the technical apparatus is sound. PRISMA-guided sourcing from Scopus, a documented data-cleaning pipeline (Python with pandas and NLTK, a custom synonym dictionary, manual correction of lemmatization artifacts), a keyword co-occurrence network in VOSviewer, Louvain clustering executed across 100 random seeds, and cosine-similarity article-to-cluster mapping together constitute a defensible and relatively rigorous workflow.

 Two design issues should be addressed:

- **The "systematic review" of 30 articles is not executed as a systematic review.** A systematic review requires explicit eligibility criteria, a screening and appraisal process, and a structured synthesis. What is presented is a set of representative/illustrative articles per cluster and era (Table 2). A prior reviewer specifically requested a comprehensive table covering all aspects of these studies, which is not provided. The authors should either (a) commit to a genuine systematic review, with eligibility criteria, quality appraisal, and a complete data-extraction table covering all 30 studies; or (b) reframe these articles as "representative" or "exemplar" articles and remove all "systematic review" language. Option (b) is likely the more honest and straightforward fix given the study's actual aims. **This must be addressed.**
- **The LLM-assisted interpretation step is under-specified.** The use of ChatGPT-4 in an "iterative reflection process" to improve thematic clarity needs to be described transparently: exactly what task was given to the model, at what stage, what its outputs were, and what independent human verification was applied to prevent the model from imposing or hallucinating themes. As currently written, this is both a transparency and a validity concern. **This should be addressed** to make the design auditable.

3. Are sufficient details of methods and analysis provided to allow replication by others? — **Partly**

 Many elements are well-documented: software versions, the iterative search-term strategy, the clustering algorithm and its number of runs, and the cosine-similarity mapping rationale. The full search strategy is provided as extended data via Zenodo.

 Replication is nonetheless limited in two specific ways that **must be addressed**:

- **Selection of the 30 key articles is not reproducible.** The stated basis — "the balance of cosine similarities, piecewise regression by era, citation metrics, and thematic alignment of keywords" — is qualitative and unquantified. The authors should specify the concrete decision rules: how many articles per cluster and per era, what cosine-similarity or citation thresholds were applied, whether selection was independent and how disagreements were resolved.
- **Manual selection of the "optimal" clustering solution.** The authors ran Louvain clustering 100 times and selected the optimal solution "based on alignment with expected thematic structures." Selecting against expected structures risks confirmation bias and is difficult to reproduce. The authors should report the quantitative criteria used (e.g., modularity scores across runs, stability of cluster assignments) and clarify how "expected thematic structures" were defined a priori.

Providing the resolved cluster assignments and the article-selection log as supplementary material would substantially strengthen replicability.

 4. If applicable, is the statistical analysis and its interpretation appropriate? — **Partly**

 The piecewise linear regression is an appropriate choice for identifying inflection points in publication output, and the reported fit (R² = .9962) is excellent. Table 1's era-by-era bibliometric summary is informative and clearly presented.

 The interpretation requires moderation in one specific respect that **should be addressed**:

- **Declining Era4 citation rates.** The paper interprets the lower average citations per document in 2018–2024 as possibly reflecting "thematic saturation." This is largely a recency artifact: recent publications have had less time to accrue citations, a well-known feature of bibliometric data. The authors should foreground this artifact as the primary explanation and present "saturation" only as a secondary, speculative possibility, or remove it. A standard normalization (e.g., citations per year, field-normalized indicators, or restricting comparison to a fixed citation window) would make any saturation claim more defensible.

A note of caution: bibliometric counts describe attention and output, not mechanism or efficacy. Some interpretive statements drift toward substantive claims and should be tempered (see Question 6).

 5. Are all the source data underlying the results available to ensure full reproducibility? — **Partly**

 The authors state that no data are associated with the article but provide the bibliometric search strategy (Appendix A) as extended data on Zenodo under CC-BY 4.0, which is commendable.

 However, for a bibliometric study of this kind, full reproducibility requires more than the search strategy. **To strengthen this, the authors should deposit**: the list of the 16,585 Scopus record identifiers (or the exported metadata, subject to Scopus terms), the synonym dictionary used in cleaning, the final keyword co-occurrence matrix or network file, and the cluster assignments for each article. With the search query alone, an independent researcher cannot reconstruct the cleaned dataset or verify the cluster structure. Because the records derive from a proprietary database, the authors may not be able to share full metadata, but at minimum the record identifiers, processing scripts, and derived network/cluster files should be made available. **Providing the derived analytical files should be addressed** to support reproducibility.

 6. Are the conclusions drawn adequately supported by the results? — **Partly**

 The central descriptive conclusions — rapid growth since 2018, a five-cluster intellectual structure, the dominance of clinical/therapeutic research, and geographic concentration in the US and Europe — are well-supported by the analysis.

 Two conclusions over-reach the evidence and **should be addressed**:

- **Cultural-bias claim.** The inference that underrepresentation of Africa and South Asia reflects a "cultural bias" in the field is partly circular: an English-only, Scopus-only design will itself underrepresent non-English and regionally indexed literature. The authors should explicitly acknowledge, at the point where this finding is reported (not only in the limitations), that the observed geographic pattern is partly a product of the data sources, and temper the claim accordingly.
- **Practice and mechanism statements.** Several conclusions about clinical efficacy, mechanisms, and nursing implementation are drawn from the cluster narratives rather than from the bibliometric data itself. Bibliometrics maps what has been studied and cited, not whether interventions work. These statements should be clearly attributed to the underlying primary literature rather than presented as findings of this bibliometric study, and causal/mechanistic language should be softened.

Recommendation

 The study addresses a worthwhile question with a substantial dataset and a credible analytical pipeline, and its thematic mapping is a useful contribution. In its current form, however, the manuscript has accuracy, transparency, and reproducibility gaps that should be resolved before it can be considered fully scientifically sound. I would categorize this as **approved with reservations**, with the expectation that the five essential points above are addressed in revision.

Is the work clearly and accurately presented and does it cite the current literature?

Partly

If applicable, is the statistical analysis and its interpretation appropriate?

Partly

Are all the source data underlying the results available to ensure full reproducibility?

Partly

Is the study design appropriate and is the work technically sound?

Partly

Are the conclusions drawn adequately supported by the results?

Partly

Are sufficient details of methods and analysis provided to allow replication by others?

Partly

Reviewer Expertise:

Sleep, Mental Health, Nursing, Community, Public Health, Depression

I confirm that I have read this submission and believe that I have an appropriate level of expertise to confirm that it is of an acceptable scientific standard, however I have significant reservations, as outlined above.

# Reviewer report: Reviewer response for version 2

**DOI:** 10.5256/f1000research.197699.r472120

**Published:** 2026-04-02

**Authors:** Ek-uma Imkome

**Affiliations:** 1 Independent Scholar, Pratumtani, Thailand

I appreciate your thorough responses to the critical points. The revisions adequately address my concerns and improve the manuscript. I have no further comments and hope that this bibliometric analysis contributes meaningfully to nursing science and nursing practice.

Is the work clearly and accurately presented and does it cite the current literature?

Yes

If applicable, is the statistical analysis and its interpretation appropriate?

Yes

Are all the source data underlying the results available to ensure full reproducibility?

No source data required

Is the study design appropriate and is the work technically sound?

Yes

Are the conclusions drawn adequately supported by the results?

Yes

Are sufficient details of methods and analysis provided to allow replication by others?

Yes

Reviewer Expertise:

Mental Health and Psychiatric Nursing

I confirm that I have read this submission and believe that I have an appropriate level of expertise to confirm that it is of an acceptable scientific standard.

# Reviewer report: Reviewer response for version 1

**DOI:** 10.5256/f1000research.185982.r429569

**Published:** 2025-12-24

**Authors:** Zartashia Kynat Javaid

**Affiliations:** 1 Government College University Faisalabad, Faisalabad, Punjab, Pakistan

This paper is a good effort to synthesize data on music and wellbeing. There are recommendations for clarity and improvement.

 Abstract lacks method details.

 Introduction is brief,incomplete and structured poorly.Define wellbeing,draw link of study variables alongwith conceptual underpinnings as base of objectives and research questions clearly stated. Give rationale for study.

 Method indicate systematic review was conducted on 30 studies but abstract has no mention. Further, make comprehensive table of these studies covering all aspects.

 Discussion should based on objectives in light of literature.

Is the work clearly and accurately presented and does it cite the current literature?

Partly

If applicable, is the statistical analysis and its interpretation appropriate?

Partly

Are all the source data underlying the results available to ensure full reproducibility?

No

Is the study design appropriate and is the work technically sound?

No

Are the conclusions drawn adequately supported by the results?

No

Are sufficient details of methods and analysis provided to allow replication by others?

No

Reviewer Expertise:

Applied Psychology

I confirm that I have read this submission and believe that I have an appropriate level of expertise to confirm that it is of an acceptable scientific standard, however I have significant reservations, as outlined above.

# Response

**Published:** 2026-03-12

**Authors:** faizul hasan

**Affiliations:** faculty of nursing, Chulalongkorn University, Bangkok, Bangkok, Thailand

This paper is a good effort to synthesize data on music and wellbeing. There are recommendations for clarity and improvement.

 Response: Thank you for your acknowledgment. A comprehensive, itemized reply to each comment is presented below. Any modifications have been marked in red in the manuscript for convenient reference. We trust that these improvements sufficiently resolve your concerns to enhance the overall quality of the manuscript. Thank you once more for your invaluable feedback and consideration.

 Abstract lacks method details.

 Response: We have expanded the Methods section of the Abstract.

 Introduction is brief, incomplete and structured poorly. Define wellbeing, draw link of study variables along with conceptual underpinnings as base of objectives and research questions clearly stated. Give rationale for study.

 Response: We have substantially revised the Introduction section.

 Method indicate systematic review was conducted on 30 studies but abstract has no mention. Further, make comprehensive table of these studies covering all aspects.

 Response: We thank the reviewer for identifying this inconsistency between the Methods section and the Abstract. We have addressed this issue.

 Discussion should based on objectives in light of literature.

 Response: The Discussion is now structured around the three research objectives, with each section explicitly interpreting findings in relation to the broader literature. Implications for research, clinical practice, and policy have been added.

# Reviewer report: Reviewer response for version 1

**DOI:** 10.5256/f1000research.185982.r421932

**Published:** 2025-10-24

**Authors:** Ek-uma Imkome

**Affiliations:** 1 Independent Scholar, Pratumtani, Thailand

**Title of Article**

*A Bibliometric Analysis of Music's Role in Promoting Well-Being in Health Science Research*

 This manuscript presents a timely and methodologically rigorous bibliometric synthesis of global research on music and well-being. The authors employ Louvain clustering, cosine similarity, and PRISMA-guided data collection to produce a comprehensive overview of more than 16,000 articles. The analysis is distinguished by its clarity and rigor, effectively presenting complex data in a well-structured and accessible format. The thematic organization is coherent, and the discussion thoughtfully highlights the role of nurses in music-based interventions, which aligns well with the journal’s interdisciplinary scope.

 This work makes a meaningful contribution to the literature and provides a strong foundation for future cross-sector collaboration.

**Minor Suggestions for Improvement**

 1. **Abstract Enhancement**

- Clarify the study’s objectives and provide a concise summary of key findings (e.g., number of clusters, leading countries, prominent trends).
- Include explicit implications for practice or policy to strengthen the abstract’s relevance for clinicians and educators.

2. **Nursing Integration**

- Expand the discussion on how music-based approaches can be integrated into nursing education and clinical practice.
- Consider adding a dedicated subsection detailing nursing implications to enhance applicability for healthcare professionals.

3. **Limitations and Future Directions**

- The limitations section is well-articulated but could benefit from quantifying the potential bias due to language or database scope.
- Future directions could be more specific, such as proposing cross-cultural collaborations or ethical frameworks for digital music therapy.

The authors are to be commended for the manuscript’s clarity, methodological rigor, and interdisciplinary relevance. This work represents a valuable contribution to the field and offers a strong foundation for future research and collaboration.

 I wish the authors continued success in their academic endeavors.

Is the work clearly and accurately presented and does it cite the current literature?

Yes

If applicable, is the statistical analysis and its interpretation appropriate?

Yes

Are all the source data underlying the results available to ensure full reproducibility?

No source data required

Is the study design appropriate and is the work technically sound?

Yes

Are the conclusions drawn adequately supported by the results?

Yes

Are sufficient details of methods and analysis provided to allow replication by others?

Yes

Reviewer Expertise:

Mental Health and Psychiatric Nursing

I confirm that I have read this submission and believe that I have an appropriate level of expertise to confirm that it is of an acceptable scientific standard, however I have significant reservations, as outlined above.

# Response

**Published:** 2026-03-12

**Authors:** faizul hasan

**Affiliations:** faculty of nursing, Chulalongkorn University, Bangkok, Bangkok, Thailand

This manuscript presents a timely and methodologically rigorous bibliometric synthesis of global research on music and well-being. The authors employ Louvain clustering, cosine similarity, and PRISMA-guided data collection to produce a comprehensive overview of more than 16,000 articles. The analysis is distinguished by its clarity and rigor, effectively presenting complex data in a well-structured and accessible format. The thematic organization is coherent, and the discussion thoughtfully highlights the role of nurses in music-based interventions, which aligns well with the journal’s interdisciplinary scope.

 This work makes a meaningful contribution to the literature and provides a strong foundation for future cross-sector collaboration.

 Response: Thank you for your recognition. A detailed, itemised response to each comment is provided below. Any changes have been highlighted in red in the manuscript. We believe that the improvements adequately address your comments to improve the manuscript's overall quality. Thank you again for your invaluable feedback and consideration.

 Minor Suggestions for Improvement

 1. Abstract Enhancement

 Clarify the study’s objectives and provide a concise summary of key findings (e.g., number of clusters, leading countries, prominent trends).

 Include explicit implications for practice or policy to strengthen the abstract’s relevance for clinicians and educators.

 Response: We have revised this section accordingly.

 2. Nursing Integration

 Expand the discussion on how music-based approaches can be integrated into nursing education and clinical practice.

 Consider adding a dedicated subsection detailing nursing implications to enhance applicability for healthcare professionals.

 Response: This section has been updated in accordance with the aforementioned.

 3. Limitations and Future Directions

 The limitations section is well-articulated but could benefit from quantifying the potential bias due to language or database scope.

 Future directions could be more specific, such as proposing cross-cultural collaborations or ethical frameworks for digital music therapy.

 Response: We have revised it.

 The authors are to be commended for the manuscript’s clarity, methodological rigor, and interdisciplinary relevance. This work represents a valuable contribution to the field and offers a strong foundation for future research and collaboration.

 I wish the authors continued success in their academic endeavors.

 Response: Thank you again for your acknowledgment.
